# Synergistic interaction between praziquantel and oxfendazole or fenbendazole in a murine cysticercosis model

**DOI:** 10.1128/aac.00560-25

**Published:** 2025-08-18

**Authors:** Palomares-Alonso Francisca, González-Maciel Angélica, Reynoso-Robles Rafael, Castro Nelly, Pérez-Severiano Francisca, Sánchez-Mendoza Alicia, Jung-Cook Helgi, Bravo Guadalupe, López-Muñoz Francisco

**Affiliations:** 1 Laboratorio de Neuroinflamación, Instituto Nacional de Neurología y Neurocirugía, Ciudad de México, Mexico; 2 Laboratorio de Morfología Celular y Tisular, Instituto Nacional de Pediatría 37759, Ciudad de México, Mexico; 3 Laboratorio de Neurobioquímica y Conducta, Instituto Nacional de Neurología y Neurocirugía 61614 , Ciudad de México, Mexico; 4 Laboratorio de Neurofarmacología molecular y nanotecnología, Instituto Nacional de Neurología y Neurocirugía 61614 , Ciudad de México, Mexico; 5 Departamento de Farmacología, Instituto Nacional de Cardiología 61588 , Ciudad de México, Mexico; 6 Facultad de Química, Departamento de Farmacia, Universidad Nacional Autónoma de México, Ciudad de México, Mexico; 7 Departamento de Farmacobiología, Cinvestav-Sede Sur 505469 , Ciudad de México, Mexico; The Children's Hospital of Philadelphia, Philadelphia, Pennsylvania, USA

**Keywords:** murine cysticercosis, oxfendazole, fenbendazole, praziquantel and synergistic effect

## Abstract

Up to date, the pharmacological treatment for active neurocysticercosis (NCC) includes only two drugs, praziquantel and albendazole; however, the clinical response is not always successful, due to its low bioavailability; therefore, new approaches are needed. The aim of the present study was to evaluate the pharmacological interaction between praziquantel (PZQ) and oxfendazole (OXF) *in vitro*; also, the drug-drug interaction between PZQ and OXF or fenbendazole (FBZ) *in vivo* using *Taenia crassiceps* metacestode was assessed. For the *in vitro* study, the surface of synergistic interaction (SSI) analysis was used to determine the kind of drug interaction between PZQ and OXF. Additionally, morphological and ultrastructural effects were investigated using transmission scanning electron microscopy. For the *in vivo* study, different doses of PZQ and FBZ or OXF were combined, and the cysticidal effect was determined. *In vitro,* results showed that 12 combinations exhibited synergism, and four exhibited additive effects. The 1:5 ratio of PZQ and OXF exhibited the highest synergistic effect, with an 11-fold increase in comparison to their theoretical sum. Microscopic observations revealed damage to the tegument and germinal layer; microtriches, flame cells, and muscular fibers were also affected. *In vivo,* results showed that PZQ + FBZ and PZQ + OXF (ratios 1:4 and 1:1, respectively) exhibited a synergistic reduction of the parasites. The findings of the present study show the potential of these combinations as a pharmacological alternative for cysticercosis treatment. Complementary studies are needed to determine their benefits in the clinical field.

## INTRODUCTION

Neurocysticercosis (NCC), caused by porcine tapeworm cysticerci, *Taenia solium*, is one of the most lethal parasite infections of the central nervous system. This disease is endemic in many countries in Latin America, Africa, and Asia (1). Recently, some cases have been found in non-endemic countries such as the United States and Australia (2, 3). NCC represents a public health problem, and it is the most common cause of acquired seizures and epilepsy, mainly in developing countries (4). NCC treatment depends on the number, localization, and stage of the evolution of the parasite. Pharmacological treatment with anthelmintic drugs is recommended when the cysts are viable. Praziquantel (PZQ) and albendazole (ABZ) are the drugs of choice for this intervention. For ABZ, a dose of 15 mg/kg is recommended, and for PZQ, a dose of 50 mg/kg is usually used (5). However, none of them is 100% effective, which has been related to their low bioavailability and their extensive first-pass hepatic metabolism to inactive metabolites (6, 7). Different approaches have been assessed to find new therapeutic options for cysticercosis treatment, including the combined treatment of drugs.

ABZ (15 mg/kg/day) in combination with PZQ (50 mg/kg/day) has been proposed as an effective therapy for patients with >2 viable parenchymal cysticerci (8). Garcia et al. showed that the combined treatment increased the complete resolution of cysts in 64% of patients with intraparenchymal neurocysticercosis, whereas with the standard ABZ treatment (15 mg/kg), only 37% of the patients presented a complete resolution of cysts. Nevertheless, the treatment with ABZ at a dose of 22.5 mg/kg showed similar efficacy (53%) to that observed with the combination. Also, no significant differences in adverse events were reported between treatment groups (8).

Oxfendazole (OXF), which has a similar structure to ABZ, is a broad-spectrum anthelmintic that is currently marketed for use against lungworms and enteric helminths in beef livestock. Approved for veterinary use, OXF has been recommended as an additional strategy for control programs, including giardiasis in dogs and roundworms in horses and cattle (9). Recently, the efficacy of OXF (30 mg/kg) formulations against cysticercosis in naturally infected pigs was evaluated. Results showed a 100% efficacy against cysts located in the muscle, heart, and tongue. Likewise, OXF attained an efficacy of 65% against cysts in the brain (10). After oral administration, OXF is metabolized to metabolites fenbendazole sulfone and fenbendazole (FBZ) (11).

Also, recent work demonstrated the safety and bioavailability of OXF in healthy human volunteers (NCT02234570, NCT03035760), enhancing the possibility of a new and needed option for the treatment of helminths and tissue parasites such as neurocysticercosis (11). The PZQ + OXF (40 mg/kg + 30 mg/kg) has been evaluated against echinococcosis in sheep and was effective in killing the hydatid cysts (12), providing the usefulness of the combination for the treatment of systemic infections.

Considering this information, the main objectives of the present study were to assess the *in vitro* activity of PZQ + OXF and the *in vivo* cysticidal effect produced by PZQ + OXF and PZQ + FBZ. For the *in vitro* study, a surface of synergistic interaction (SSI) analysis was used to determine the kind of drug interaction between PZQ and OXF (13). Also, morphological and ultrastructural effects were investigated using transmission scanning electron microscopy. For the *in vivo* study, different doses of PZQ and FBZ or OXF were combined, and the cysticidal effect was determined using the experimental murine cysticercosis model.

## MATERIALS AND METHODS

### Drugs and reagents

PZQ, FBZ, and OXF were purchased from Sigma-Aldrich Chemical Co. (St. Louis, MO, USA). The culture medium used for the cysticidal assays was Dulbecco’s modified Eagle’s medium with high glucose content (DMEM, Sigma-Aldrich Co., St. Louis, MO, USA), supplemented with 10% fetal calf serum, 2 mM L-glutamine, 8 mg/dL of gentamicin sulfate, and 200,000 IU/dL of penicillin G sodium (Gibco, Gran Island, NY, USA). Carboxymethylcellulose (CMC; Golden Bell, México) and dimethyl sulfoxide (DMSO; Merck, Schuchardt, Germany; 99% assay) were analytical reagent grade.

### Parasites

*Taenia crassiceps* cysticerci (ORF strain) were obtained from experimentally infected female BALB/c mice, according to a previously described procedure (14). The study was reviewed and approved by the Institutional Committee for Handling and Animal Care of the Instituto Nacional de Neurología y Neurocirugía Manuel Velasco Suarez (No. 51/15) and was carried out according to the Mexican Guidance (NOM-062-ZOO-1999). For experiments, only those cysts sized 2–3 mm, without budding, with translucent membrane, and exhibiting an intact bladder surface were used.

### *In vitro* studies

The *in vitro* evaluation consisted of two sets of experiments. In the first set, the activity of PZQ and OXF was evaluated to obtain concentration-response curves. In the second set, the drugs were combined at different ratios to analyze possible synergistic interactions.

#### Single drug evaluation

*In vitro* cysticidal activity was evaluated for each drug. A stock solution of OXF (1 mg/mL) was prepared in DMSO, while for PZQ, a stock solution of 1 mg/mL was prepared in ethanol. The stock solutions of each drug were serially diluted to prepare working solutions in DMEM to obtain concentrations for PZQ from 20 to 200 nM, and for OXF from 300 to 3,800 nM. DMSO concentration did not exceed 0.25% or ethanol 0.1% in culture medium. Solutions of 0.25% DMSO and 0.1% ethanol in DMEM were used as negative controls. Twenty-four well cell culture flat-bottom microplates (NUNC, Denmark) were filled with 2 mL of culture medium containing each drug, 0.25% of DMSO, or 0.1% ethanol. Ten cysts were placed into each well and incubated at 37°C with a 5% CO_2_ atmosphere and 98% relative humidity for 11 days. The culture medium was changed every two days. Each experiment was performed in triplicate. Parasites were observed every day and monitored for their integrity, motility, morphological aspect, and mortality using an inverted light microscope ICM 405 (Carl Zeiss Inc., USA). The criteria to assess parasite mortality were loss of vesicular fluid, membrane paralysis, and parasite collapse. Mortality was confirmed on day 12, using the trypan blue exclusion test (15). Mortality results were analyzed using nonlinear regression to obtain the corresponding EC_50_ (effective concentration that killed 50% of the cysts) and the confidence limits for each drug.

#### Drug combination evaluation

To observe a synergistic effect, the drug’s concentrations eliciting mortality at a rate lower than 50% for each drug alone were selected. Sixteen combinations were included in the study. The range of concentrations was 30 to 150 nM for PZQ and 300 to 730 nM for OXF. DMEM with 0.25% of DMSO and 0.1% of ethanol was used as a negative control. The incubation conditions and the procedure to assess mortality were the same as those described in the single drug evaluations.

To analyze the interaction of PZQ and OXF combination, the SSI was constructed. This analysis is used to assess the pharmacological effect produced by the drugs individually, or with different ratios in combination, and allows the determination of the optimal ratio that produces synergistic cysticidal activity. First, a three-dimensional graph using the mean mortality produced by each drug concentration, either alone or in combination, was constructed. After the cysticidal effects produced by the different combinations were subtracted from the individual effects, and finally, all the planes were joined to obtain a surface. Results higher than 0 indicated potentiation, whereas those at the “0” level indicated addition (13).

#### Evaluation by microscopy

The effect of PZQ 147 nM + OXF 313 nM (1:2 ratio) on the parasite tissue was evaluated considering its *in vitro* high synergism. The cysts exposed to single treatments were also evaluated. Cysts were observed on day 12, and morphological changes, including alterations in shape and size, were observed by inverted light microscopy using an ICM 405 microscope (Carl Zeiss Inc., USA). Also, observations at the ultrastructural level were made by transmission electron microscopy (TEM). Cysts were washed with 0.9% saline solution, fixed with 2.5% glutaraldehyde (1 h), rinsed with 0.1 M phosphate buffer solution (PBS, pH 7.4), post-fixed in 0.5% osmium tetroxide (Merck, Germany), dehydrated in a graded ethanol series, and embedded in epoxy resin (Electron microscopy Sciences, PA, USA). Subsequently, resin polymerization was carried out at 60°C overnight, and then sections were cut using an ultramicrotome (Leica EM UC6, Österreich, Austria). To select areas for ultrastructural analysis, sections of 1 µm thickness were cut, placed on glass slides, stained with 1% toluidine blue, and examined using a Zeiss Axioscop-2 Plus microscope (AxioCam MRc, Jena, Germany). Once selected, the best areas for TEM, ultra-thin sections of 60 nm, were cut and recovered on coated Formvar copper grids, stained with 3% uranyl acetate/0.3% lead citrate, and examined using a JEOL 1011 microscope at 70 kV. Electronic microscopy photographs were taken. Addition of scale bars, lettering, and arrows on photographs was performed using Adobe Photoshop CS3 Extended (Version 10.0).

### *In vivo* cysticidal activity

Considering that OXF as well as FBZ exhibit anthelmintic activity, FBZ was also included for the *in vivo* study (16, 17).

#### Preparation of drug dispersions for treatments

Different doses of drugs were administered orally as liquid dispersion. Drugs were dispersed in 0.5% (wt/vol) CMC at different concentrations. For drug combinations, each drug was previously dispersed in 0.5% (wt/vol) CMC and then mixed. A solution of CMC 0.5% was used as a negative control.

#### Experimental infection

The study was conducted using the *T. crassiceps* murine cysticercosis model. The experimental conditions of infection and pharmacological treatment were selected based on a previous study performed by our group (14). Briefly, female BALB/c mice weighing 16–18 g were infected with 30 cysts of *T. crassiceps* (2 to 3 mm in diameter) per mouse, by intraperitoneal injection. Before inoculation, the integrity of the cysts was verified using a stereoscopic microscope, and for the injection, the cysts were previously suspended in 0.5 mL of sterile 0.9% saline solution. After 10 days of infection, the animals were ready for pharmacological treatments. During all experiments, mice were housed in animal facilities under controlled environmental conditions (20°C ± 2°C temperature and 55% ± 5% humidity) and a 12 h light/dark cycle. Food and water were provided *ad libitum*. The study was approved by the Institutional Committee of Research of the Instituto Nacional de Neurología y Neurocirugía Manuel Velasco Suárez (registration number 51/15) as well as by the Instituto Nacional de Pediatría 2023/C-002, and was carried out according to Mexican Guidance (NOM-062-ZOO-1999).

#### Drug administration

The study included two stages: in the first stage, the concentration-response curve for each drug alone was performed to select the doses for the drug combination, and in the second stage, the evaluations of the combinations were assessed.

#### Single drug evaluation

Sixty-six mice infected with *T. crassiceps* cysts were divided into eleven groups of six animals which received the following treatments: (i) PZQ 15 mg/kg, (ii) PZQ 25 mg/kg, (iii) PZQ 30 mg/kg, (iv) PZQ 50 mg/kg, (v) PZQ 100 mg/kg, (vi) OXF 15 mg/kg, (vii) OXF 25 mg/kg, (viii) OXF 50 mg/kg, (ix) OXF 100 mg/kg, (x) OXF 200 mg/kg, (xi) FBZ 15 mg/kg, (xii) FBZ 25 mg/kg, (xiii) FBZ 50 mg/kg, (xiv) FBZ 100 mg/kg, (xv) FBZ 200 mg/kg, and (xvi) a solution of 0.5% CMC in water (control group). Each mouse received a volume of 0.1 mL/20 g body weight, intragastrically, during 20 consecutive days. On day 21, the mice were sacrificed by cervical dislocation, and the cysts were carefully removed from the peritoneal cavity. The parasites were washed with sterile 0.9% saline solution and observed microscopically. Afterward, they were homogenized in a Poly-Tron PT 2100 homogenizer (Kinematica, Switzerland), then the material was centrifuged for 20 min at 5,000 rpm, and the residue was lyophilized for 4 h. Parasite residue weight was recorded using an analytical balance (Mettler 2500). The cysticidal efficacy was expressed as the mean weight of cysts recovered after treatment and was calculated by comparing the dry weight of treated experimental animals with that obtained from infected control mice.

#### Drug combination evaluation

The doses selected for the combinations were those that exhibited less than 50% efficacy to detect a possible additive or synergistic effect. Mice infected with *T. crassiceps* cysts (10 days of infection) were divided into groups of six mice; a control group receiving 0.5% CMC solution was included. The groups included were (i) control, (ii) PZQ (25 mg/kg), (iii) OXF (25 mg/kg), (iv) FBZ (100 mg/kg), (v) PZQ + OXF, and (vi) PZQ (25 mg/kg) + FBZ (100 mg/kg). Each mouse received 0.1 mL/20 g body weight of the drug dispersion or 0.5% CMC solution. Drug dispersions were applied intragastrically during 20 consecutive days. On day 21, mice were sacrificed by cervical dislocation, and cysts were carefully removed from the peritoneal cavity. Parasites were processed for dry weight to obtain the efficacy of each treatment as previously described in the single-drug evaluation.

### Statistical analysis

The EC_50_ of each drug (effective concentration that killed 50% of the cysts) was obtained using a four-parameter logistic equation (Hill function) (18). The *in vitro* mortality and *in vivo* efficacy results were expressed as the mean ± standard deviation of mean (SD).

For the *in vitro* drug combination, the observed mortality exhibited by the combinations was compared with the expected value using Student’s *t*-test. The mortality (%) produced by either PZQ or OXF (evaluated separately) was compared with the mortality value obtained from the corresponding combination by using analysis of variance (ANOVA) and *t*-test. The synergic effects between these drugs (Fig. 3A and B) were evaluated by a two-way ANOVA.

*In vivo* results were reported as the percentage reduction in the parasite weight obtained after the combination (PZQ + OXF EXP) and were compared with the sum of the parasite reduction obtained with each drug (PZQ + OXF SUM) using two-way ANOVA followed by a *t*-test. Statistical analysis was performed using SPSS software (Version 17.0), and differences were considered significant at *P* < 0.05. SPSS software (Version 17.0) was used for statistical analyses, and differences were considered significant at *P* < 0.05. The data were expressed as the mean ± SD of at least six animals.

## RESULTS

### *In vitro* cysticidal activity

#### Single drug activity

**Fig 1:**
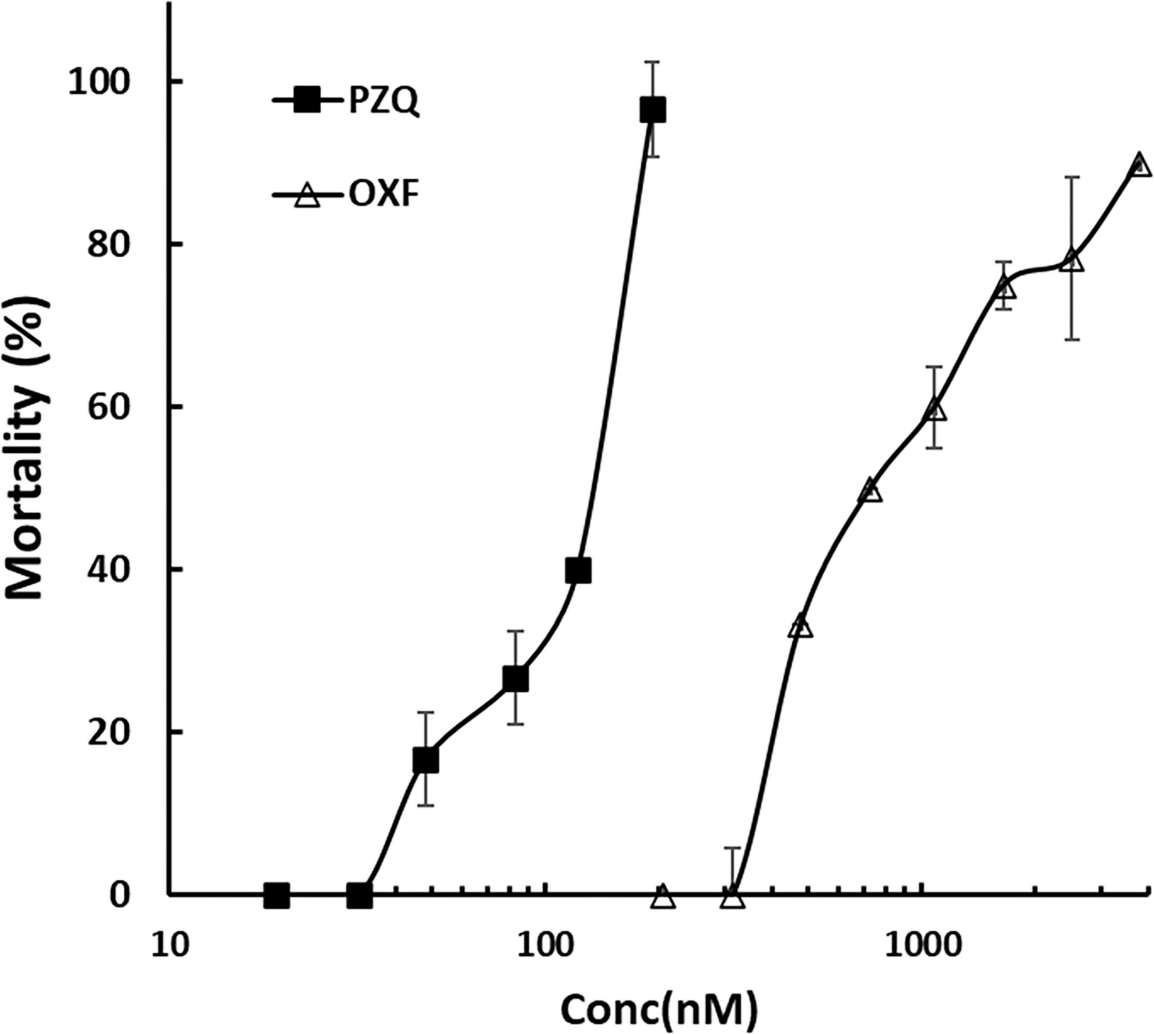
Concentration–response curves of PZQ and OXF on *Taenia crassiceps* cysts. Both drugs exhibited concentration-dependent activity. Each point represents the mean ± SD, *n* = 9.

Figure 1 shows the concentration-response curves of OXF and PZQ. The results showed that both drugs exhibited cysticidal activity in a concentration-dependent manner. The EC_50_ values obtained for OXF and PZQ were 563.1 nM (398.1–782.3) and 118.5 nM (85.3–163.3), respectively. Although PZQ was the most potent, both drugs exhibited similar efficacy.

#### *In vitro* drug combination

**Fig 2:**
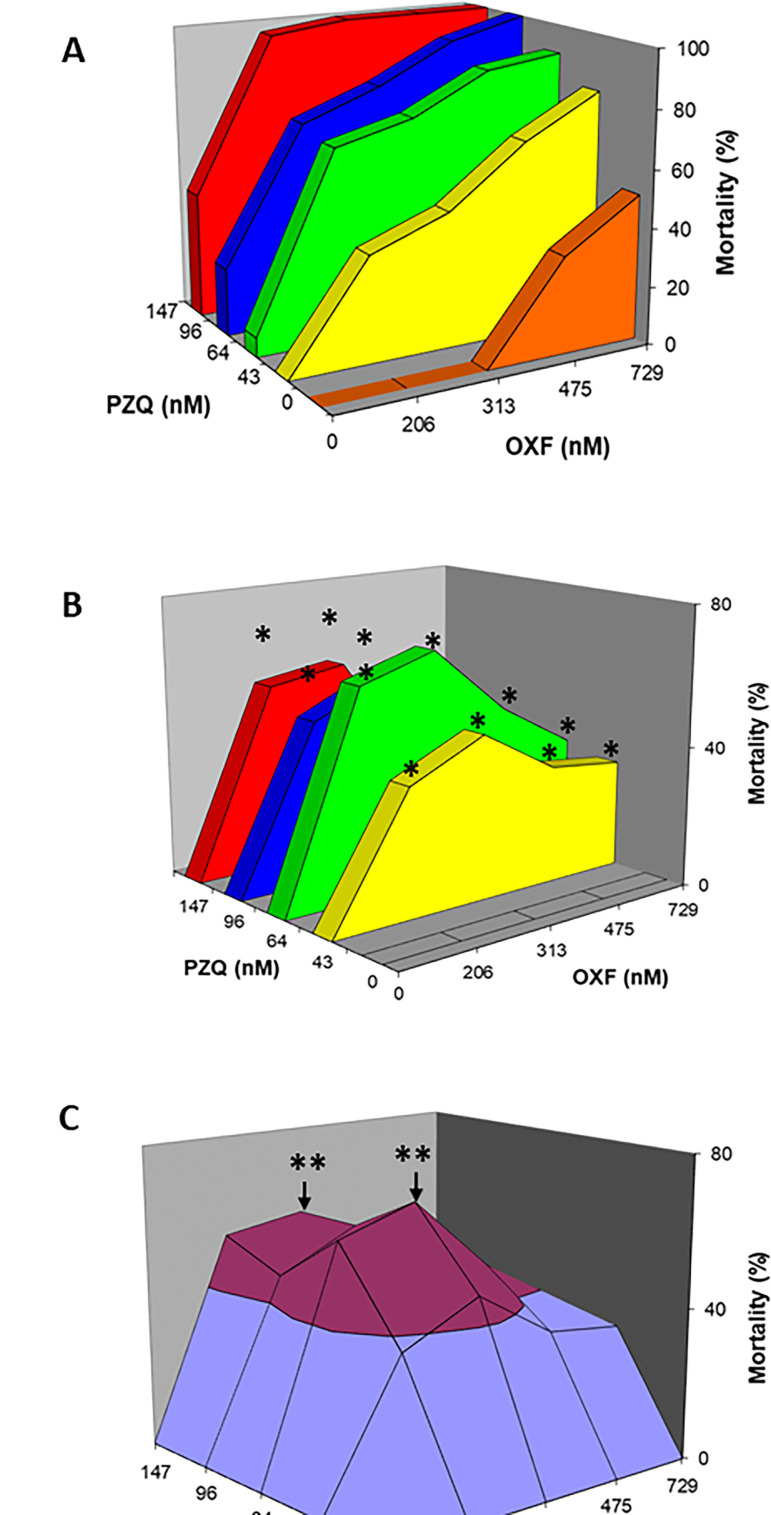
(**A**) Cysticidal effect obtained with PZQ and OXF either alone or in combination after *in vitro* drug treatment. The y-axis corresponds to mortality; the x-axis is the dose of OXF administered either alone or in simultaneous combination with PZQ, whereas the z-axis corresponds to the dose of PZQ either alone or in simultaneous combination with OXF. Each point represents the mean of three experiments (*n* = 9). (**B**) Cysticidal effects produced by the different combinations of PZQ and OXF after subtracting the individual effects. Twelve combinations showed various degrees of synergism (*). (**C**) SSI for PZQ + OXF. All synergistic combinations with an efficacy higher than 50% are shown in maroon color. PZQ (64 nM) + OXF (313.9 nM) and PZQ (147.2 nM) + OXF (313.9 nM) produced the optimal efficacy and synergistic effect (**).

Figure 2A shows the cysticidal effect for each of the drugs alone and each of the components in different combination ratios. The results showed that the combination of the drugs improved the cysticidal efficacy. Figure 2B was constructed to distinguish between additive and synergistic effects. In this analysis, if the sum of the corresponding individual mortality was higher than the theoretical sum, the result was considered to show synergistic effect; if it was lower than the theoretical sum, it was considered to show an infra-additive effect, and if it was similar to the theoretical sum, the result was considered to show additive cysticidal effect (13). In this analysis, 12 out of 16 combinations exhibited synergistic effects, and the other four exhibited additivity. PZQ (64 nM) + OXF (313.9 nM) produced the optimal synergistic effect. Figure 2C shows all the synergism points from panel B that have been joined by a plane. It can be seen that the combinations showed various degrees of synergism. PZQ (64 nM) + OXF (313.9 nM) and PZQ (147.2 nM) + OXF (313.9 nM) produced optimal efficacy and the best synergistic effect.

**Fig 3:**
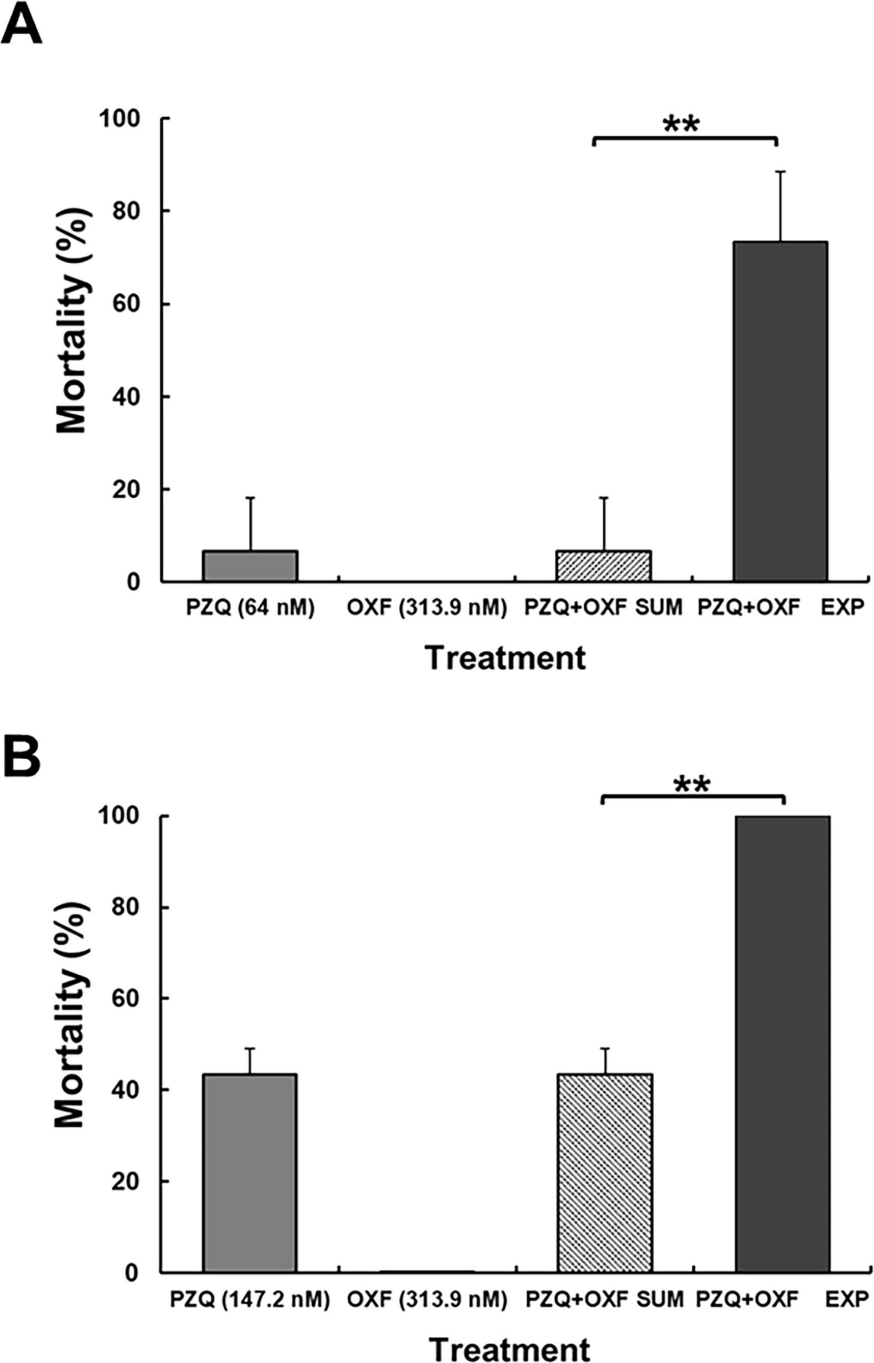
Cysticidal activity of the optimal drug combinations in comparison with each drug alone and the theoretical sum (PZQ + OXF SUM). (**A**) PZQ (64 nM) + OXF (313.9 nM) (PZQ + OXF EXP) (1:5 ratio) resulted in an increase of 11-fold in comparison to the PZQ + OXF SUM (optimal synergistic result). (**B**) PZQ (147.2 nM) + OXF (313.9 nM) (1:2 ratio) resulted in an increase of 2.3-fold in comparison to the theoretical sum. **P < 0.001 (optimal cysticidal efficacy) (two-way ANOVA followed by *t*-test). Each bar represents the mean of parasite mortality from three different experiments ± SD. *n* = 9.

Figure 3 shows the results obtained from optimal combinations. The highest synergic effect was obtained with PZQ + OXF (64 nM + 313.9 nM, 1:5 nmolar ratio), with an increase of 11 times (73.3%) in comparison to the theoretical sum of the efficacy of each drug separately (6.7%). The same cysticidal efficacy was attained with PZQ alone (192 nM) using a concentration three times higher than that used in the combination (64 nM) or with OXF alone (1,809 nM) at a concentration 5.7 times higher than that used in the combination (313.9 nM). In the case of the PZQ + OXF (147.2 + 313.9 nM, 1:2 nmolar ratio), the efficacy was 100%, whereas individually, a low effect was observed (efficacy of PZQ 147.2 nM 43.3% and OXF 313.9 nM exhibited no effect) (Fig. 3B).

#### Evaluation by microscopy

**Fig 4:**
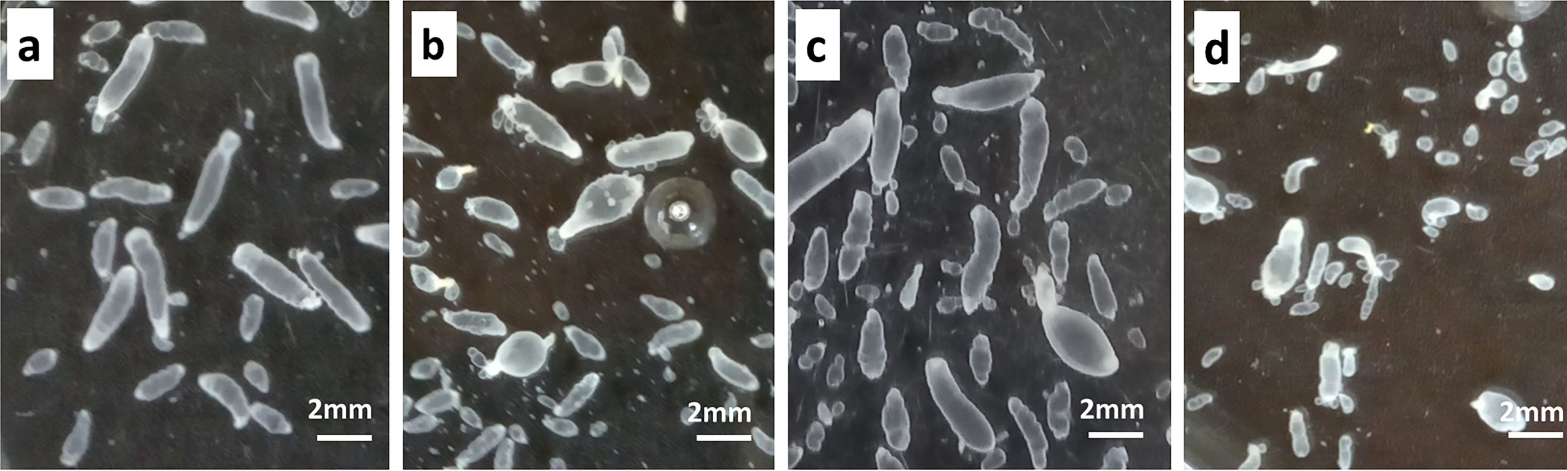
Representative microphotographs obtained from inverted light microscopy of *T. crassiceps* cysts after the *in vitro* treatment. (**a**) Parasites in the control group (0.25% DMSO) remained unaltered, (**b**) PZQ (147.2 nM), (**c**) OXF (313.9 nM), and (**d**) PZQ (147.2 nM) + OXF (313.9 nM) induced significant changes in the shape and size of cysts.

Figure 4 shows the alterations on the cysts after *in vitro* treatment with PZQ (147.2 nM), OXF (313 nM), and PZQ (147.2 nM) + OXF (313.9 nM). Cysts incubated in 0.25% DMSO remained unaltered during the experiment, exhibiting a normal appearance, with the typical alternating movements of contraction and relaxation. Parasites incubated in the presence of PZQ changed drastically in shape and size; they also lost their characteristic ruggedness and movements. OXF produced less damage to the cysts. The morphological alterations included partial loss of its motility, and some of the parasites exhibited a reduction in size that was related to the loss of vesicular fluid. Cysts incubated with PZQ + OXF exhibited more evident changes in size, shape, and motility. Some of them reduced in size as a result of the loss of vesicular fluid.

**Fig 5:**
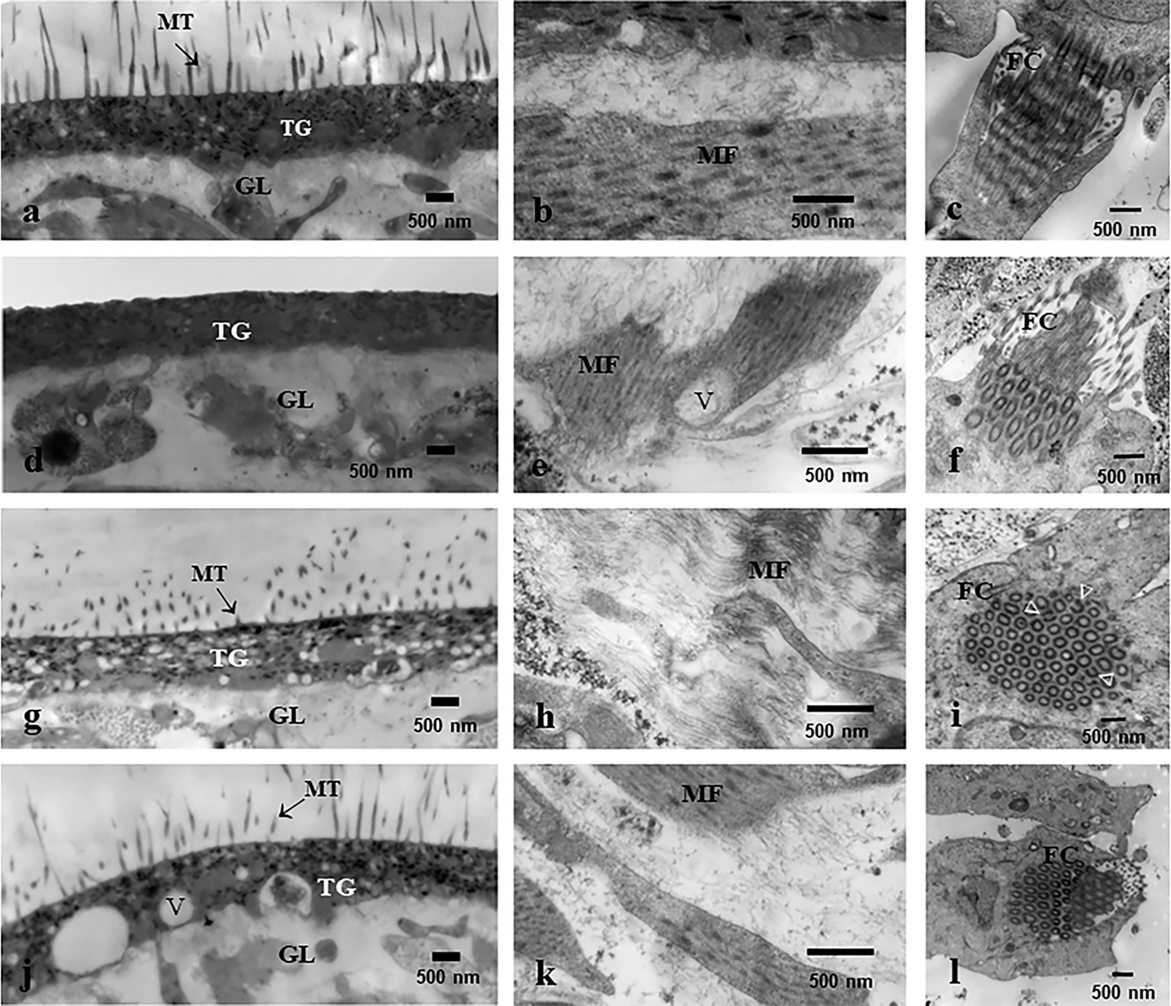
Transmission electron microscopy micrographs of *T. crassiceps* cysts after *in vitro* treatment. A representative parasite tissue is presented. (**a–c**) Cysts treated with 0.25% DMSO (control) showing tegument (TG), germinal layer (GL), microtriches (MT), muscular fibers (MF), and flame cells (FC) with normal characteristics. (**d–f**) Cysts treated with PZQ (147.2 nM) exhibit a reduction in size and number of MT, as well as the disruption of the MF and vacuoles (**V**), also visible in the germinal layer. Also, the disintegration of the typical FC architecture was observed. (**g–i**) Cysts treated with OXF (313.9 nM) display alterations in every structure. (**j–l**) Cysts treated with the PZQ (147.2 nM) + OXF (313.9 nM), a partial loss of microtriches on the tegument was observed. Also, the muscular fibers appeared disorganized in some areas.

Figure 5 shows the ultrastructural alterations on the cysts after *in vitro* treatment with PZQ (147.2 nM), OXF (313.9 nM), and PZQ (147.2 nM) + OXF (313.9 nM). In control cysts, all structures remained unaltered, including microtriches and tegument. The germinal layer, constituted by densely packed tissue containing muscular fibers, flame cells, and connective tissue, was well-conserved (Fig. 5a through c). Cysts treated with PZQ showed a loss of microtriches on the tegument (Fig. 5d), the germinal layer’s muscular fibers appeared disorganized, and in some areas, glycogen granules and vacuoles were also observed (Fig. 5e). Some flame cells showed partial disintegration of external and internal tubules, and their cytoplasm was extended (Fig. 5f). Parasites treated with OXF exhibited partial loss of microtriches (Fig. 5g). The thickness of the tegument decreased, and the muscular fibers were disorganized and broken in some areas (Fig. 5h). In some flame cells, the membrane surrounding the microtubules partially disappeared (Fig. 5i). The effect of PZQ + OXF over the cysts was more pronounced than for PZQ or OXF alone. The microtriches on the tegument were diminished in number and shorter than those observed in control parasites (Fig. 5j). In addition, destruction of the cellular integrity of the longitudinal muscular system and disruption of the muscular cells in its myofibril portion in the germinal layer were observed (Fig. 5k). The cytoplasm of the flame cells exhibited some alterations while the cilia in the inner flame were preserved (Fig. 5l).

### *In vivo* drug combinations

#### Efficacy of PZQ, OXF, and FBZ

**Fig 6:**
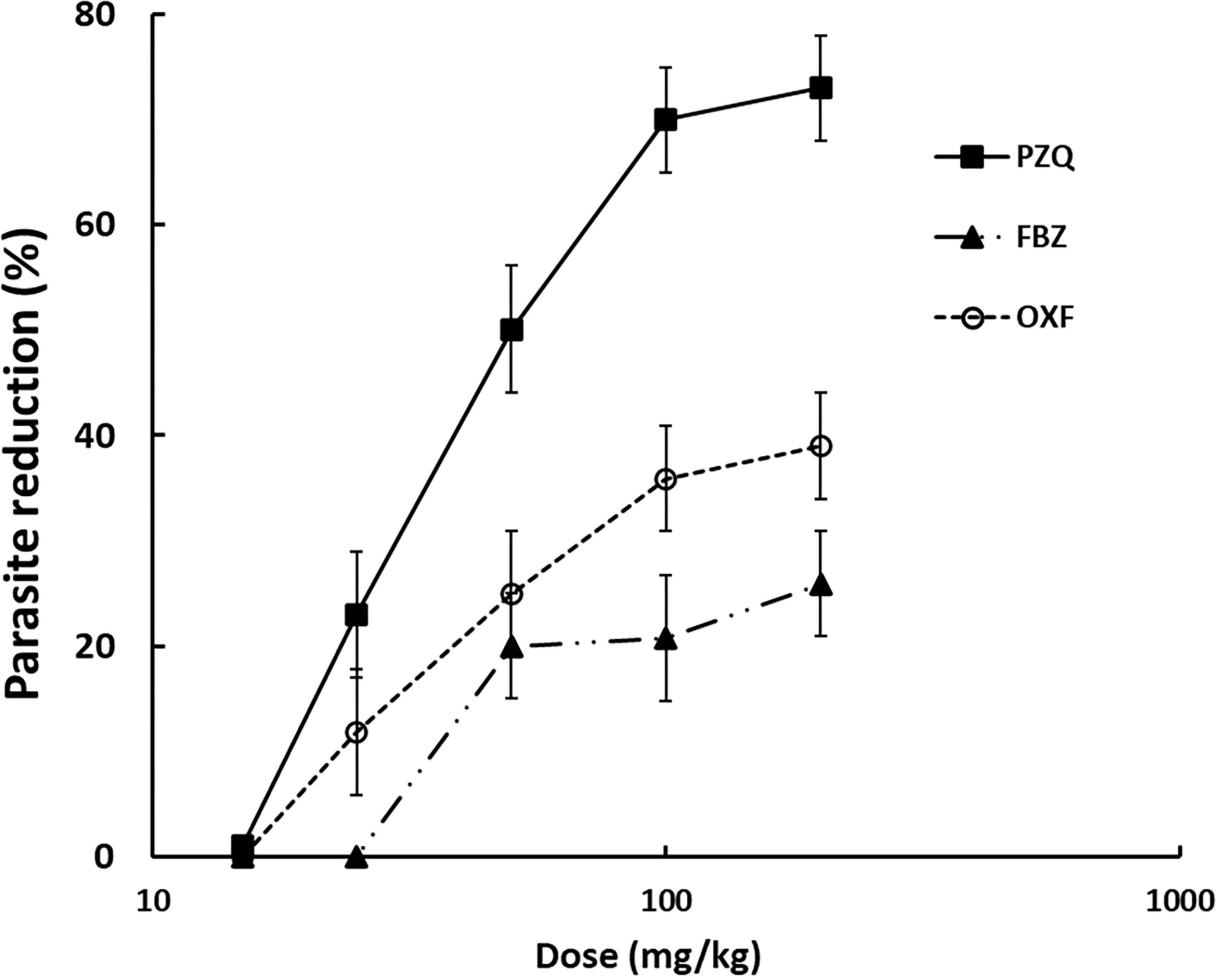
Dose-response curves of PZQ, FBZ, and OXF on murine cysticercosis by *T. crassiceps*. Each point represents the mean of the percentage effect ± SD. *n* = 6.

Figure 6 shows the *in vivo* dose-response curves to PZQ, OXF, and FBZ. All drugs showed dose-dependent cysticidal activity. FBZ and OXF showed low efficacy, exhibiting 29% and 39% load parasite decrease, respectively, at the highest dose tested (200 mg/kg), while the same dose of PZQ reached a 73% decrease in parasite load.

#### Efficacy of PZQ + FBZ and PZQ + OXF

**Fig 7:**
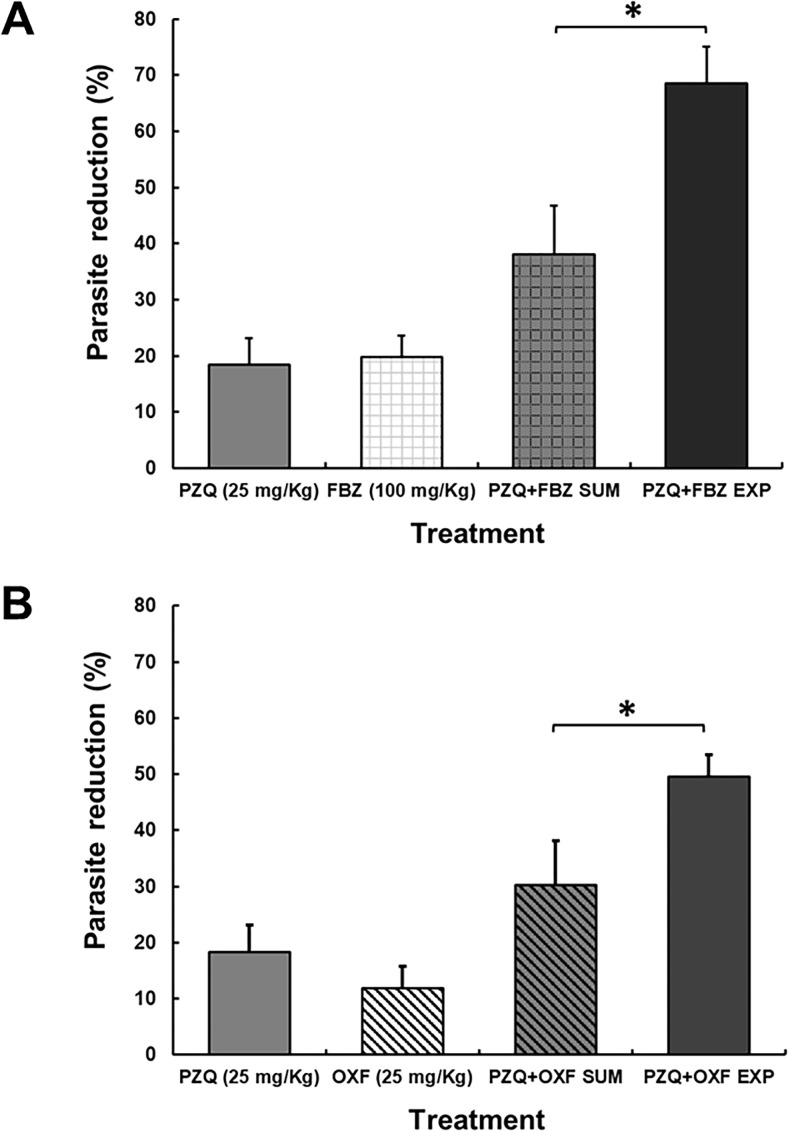
*In vivo* cysticidal effect of PZQ, FBZ, OXF, and PZQ + FBZ and PZQ + OXF (ratios 1:4 and 1:1, respectively) on murine cysticercosis by *T. crassiceps*. Each bar represents the percentage reduction in the parasite weight obtained after different treatments ± SD, *n* = 6. (**A**) PZQ (25 mg/kg) + FBZ (100 mg/kg) resulted in an increase of 1.8-fold in comparison with the sum of the parasite reduction obtained with each drug. (**B**) PZQ (25 mg/kg) + OXF (25 mg/kg) resulted in an increase of 1.6-fold in comparison to the sum of the parasite reduction obtained with each drug. **P < 0.05, two-way ANOVA followed by *t*-test.

Figure 7 shows the *in vivo* cysticidal effect of PZQ (25 mg/kg), OXF (25 mg/kg), FBZ (100 mg/kg), and the combinations against *T. crassiceps*. Figure 7A shows the cysticidal activity exerted by PZQ (25 mg/kg), FBZ (100 mg/kg), and PZQ + FBZ (25 mg/kg + 100 mg/kg). PZQ and FBZ showed a low reduction in parasite load compared to the control group (18.32% and 19.7%, respectively). In contrast, PZQ + FBZ exhibited a significant reduction in the parasite load (*P* < 0.05). A synergistic effect was observed with this combination since the experimental parasite reduction (68.5%) was higher than the sum of both drugs (38.1%) (*P* < 0.05). Figure 7B shows the results of PZQ (25 mg/kg), OXF (25 mg/kg), and PZQ + OXF (25 mg/kg + 25 mg/kg). It was observed that each drug alone reduced parasite load to 18.3% and 11.9%, respectively. The combination of drugs produced a synergistic effect observed as 49.5% parasite reduction; this result was higher than the sum of effects calculated by both drugs (30.2%) (*P* < 0.05). The doses tested for drug combinations were well tolerated, and there were no abnormalities in the behavior, food/water consumption, and general activity of the animals throughout the study.

## DISCUSSION

In the last 40 years, the pharmacological treatment for active NCC has been supported using two drugs: PZQ and ABZ; however, the results are far from ideal due to their variable efficacy, low therapeutic rate, and short half-life (1).

To find new pharmacological treatments for this disease, different strategies have been explored, such as new molecules, new formulations, and medicinal plants (14, 19, 20); however, their efficacy and safety in humans have not yet been evaluated. Another strategy for those patients who did not respond to the single treatment could be drug combinations. In this regard, Garcia et al. (8) showed that the combination of ABZ with PZQ increased the cysticidal effect of ABZ in patients with multiple brain cysts, without increasing side effects. It has been reported that ABZ shows a high inter- and intraindividual variability; therefore, it is necessary to find new options. Another drug that has been shown to have cysticidal efficacy on porcine cysticercosis is OXF (10); therefore, it was considered important to explore the cysticidal effect of OXF in combination with PZQ.

In the present work, we evaluated the *in vitro* cysticidal activity of PZQ and OXF alone and in combination against *T. crassiceps* cysts. Also, the SSI as a strategy for drug combinations was used. Additionally, *in vivo* cysticidal activity was assessed on a murine cysticercosis model.

The *in vitro* cysticidal activity exhibited by OXF against *T. crassiceps* agreed with that reported by Mkupasi et al. (21), who observed the cysticidal effect of OXF in pigs infected with *T. solium* cysts. Since the efficacy of OXF was comparable to that exhibited by PZQ, we decided to evaluate different combinations of them at different ratios. SSI showed an interesting pattern of interactions between PZQ and OXF, which depends on the ratio used and allows the determination of the optimal ratio that produces cysticidal activity. Synergism was found in 12 of 16 combinations, and another four showed an additive effect. These interactions resulted in a significant increase in the efficacy of PZQ and OXF at concentrations that exhibited very low or no effect when administered alone. The interaction could be associated with the mode of action of the drugs against *T. crassiceps* cysts, since early studies have demonstrated that PZQ disrupts calcium ion homeostasis in the helminths, and the current consensus is that it antagonizes voltage-gated calcium channels, leading to uncontrolled muscle contraction and paralysis of the parasite (22). OXF, like all benzimidazoles, exerts its effect over the formation of microtubules and affects the nutrition of the parasite (23).

The effects of treatments on the parasite structure at the ultrastructural level revealed several changes. After microscopic observation, major alterations were found with PZQ + OXF in comparison with each drug alone. The damage on the tegument and germinal layer revealed the combined effect of each drug, since the structures formed by beta-tubulin such as microtriches, flame cells, and muscular fibers were partially affected, which could be related to the effect of OXF, since the damage of the microtubule structures is related to the primary mode of action of benzimidazoles (23). In the case of PZQ, the effect was in the germinal layer, where the vacuolization of the tegument and loss of cell integrity were observed. This effect has been previously reported in the same parasite (24).

The *in vivo* results of PZQ and OXF agree with those already found in *T. crassiceps and T. solium* (21, 25). When the *in vivo* effect of FBZ was evaluated, the results showed that FBZ exhibited a low reduction in parasite load compared to OXF; however, this reduction was significant with respect to the control, confirming its anthelmintic action (26). This is the first study that evaluates the *in vivo* efficacy of FBZ and OXF against murine cysticercosis by *T. crassiceps*. The results showed that the efficacy of OXF was slightly higher than FBZ. Considering that OXF and FBZ exhibited *in vivo* cysticidal activity, it was decided to explore their combinations with PZQ. The results showed that both combinations resulted in a synergistic cysticidal effect, as the experimental efficacy of each combination was higher than the sum of the efficacy of each single drug. These results could be associated with pharmacodynamic interaction, considering the mode of action of each drug. The efficacy of a low dose of PZQ and FBZ in the management of asymptomatic schistosomiasis in dogs was recently evaluated, in which an enhancement of the efficacy of PZQ was found (26). Also, PZQ + OXF (40 mg/kg + 30 mg/kg) was shown to be effective against echinococcosis in sheep (12). In the present study, we report for the first time the synergistic effect of PZQ + OXF against *T. crassiceps*. Considering its similarity with *T. solium* in morphology, immunology, genetics, and metabolic pathways (27–29), the beneficial synergistic effect could be similar. On the other hand, OXF has demonstrated a longer plasma half-life in some animals (30, 31), suggesting that OXF might maintain effective concentrations in the body for a longer period. Additionally, the bioavailability, safety, and tolerability of OXF have been demonstrated in healthy volunteers (11, 32, 33). In the present study, the doses used for the combinations were well tolerated, and no signal of toxicity was observed in animals; however, complementary studies are needed to confirm the lack of toxicity of combinations.

### Conclusions

In the present study, the pharmacological interactions between PZQ + OXF and PZQ + FBZ were documented. The combined mode of action of drugs resulted in synergism or additivity, and these interactions increased the efficacy compared to each of the drugs individually at doses that were ineffective or with low effect. The combinations could allow for reducing the doses of the drugs without affecting the efficacy of the treatment and could contribute to reducing the adverse effects and cost of the treatment at the clinical level. Based on these results, the combinations could represent a new alternative for cysticercosis treatment. Complementary studies are needed to determine their benefits in the clinical field.

## Data Availability

Data will be made available on request.

## References

[1] Pineda-Reyes R, White AC. 2022. Neurocysticercosis: an update on diagnosis, treatment, and prevention. Curr Opin Infect Dis 35:246–254. doi:10.1097/QCO.0000000000000831 35665719

[2] Forster D, Ko D-K, Koehler AV, Kranz S, Goh C, Fleming B, Awad M, Johnson D, Gasser RB, Mahanty S. 2020. Case report: neurocysticercosis acquired in Australia. Am J Trop Med Hyg 103:2318–2322. doi:10.4269/ajtmh.20-0839 32959773 PMC7695046

[3] Spallone A, Woroch L, Sweeney K, Seidman R, Marcos LA. 2020. The burden of neurocysticercosis at a Single New York Hospital. J Pathog 2020:8174240. doi:10.1155/2020/8174240 32802516 PMC7403940

[4] Herrick JA, Bustos JA, Clapham P, Garcia HH, Loeb JA, Cysticercosis Working Group in Peru. 2020. Unique characteristics of epilepsy development in neurocysticercosis. Am J Trop Med Hyg 103:639–645. doi:10.4269/ajtmh.19-0485 32431269 PMC7410468

[5] White AC, Coyle CM, Rajshekhar V, Singh G, Hauser WA, Mohanty A, Garcia HH, Nash TE. 2018. Diagnosis and treatment of neurocysticercosis: 2017 clinical practice guidelines by the Infectious Diseases Society of America (IDSA) and the American Society of Tropical Medicine and Hygiene (ASTMH). Clin Infect Dis 66:e49–e75. doi:10.1093/cid/cix1084 29481580 PMC6248812

[6] Ochoa D, Saiz-Rodríguez M, González-Rojano E, Román M, Sánchez-Rojas S, Wojnicz A, Ruiz-Nuño A, García-Arieta A, Abad-Santos F. 2021. High-fat breakfast increases bioavailability of albendazole compared to low-fat breakfast: single-dose study in healthy subjects. Front Pharmacol 12:664465. doi:10.3389/fphar.2021.664465 33935787 PMC8082448

[7] Hong S-T. 2018. Albendazole and praziquantel: review and safety monitoring in Korea. Infect Chemother 50:1–10. doi:10.3947/ic.2018.50.1.1 29637747 PMC5895825

[8] Garcia HH, Gonzales I, Lescano AG, Bustos JA, Zimic M, Escalante D, Saavedra H, Gavidia M, Rodriguez L, Najar E, Umeres H, Pretell EJ, Cysticercosis Working Group in Peru. 2014. Efficacy of combined antiparasitic therapy with praziquantel and albendazole for neurocysticercosis: a double-blind, randomised controlled trial. Lancet Infect Dis 14:687–695. doi:10.1016/S1473-3099(14)70779-0 24999157 PMC4157934

[9] Villeneuve V, Beugnet F, Bourdoiseau G. 2000. Efficacy of oxfendazole for the treatment of giardiosis in dogs. Experiments in dog breeding kennels. Parasite 7:221–226. doi:10.1051/parasite/2000073221 11031759

[10] Arroyo G, Bustos JA, Calcina JF, Gallegos L, Vargas-Calla A, Gomez-Puerta LA, Lopez T, Gilman RH, Garcia HH, Gonzalez AE, En Cisticercosis en Perú G de T. 2021. Eficacia de dos formulaciones de oxfendazol producidas localmente para el tratamiento de la cisticercosis en cerdos infectados naturalmente. Rev Peru Med Exp Salud Publica 38:296–301. doi:10.17843/rpmesp.2021.382.6539 34468579 PMC8796694

[11] Gonzalez AE, Codd EE, Horton J, Garcia HH, Gilman RH. 2019. Oxfendazole: a promising agent for the treatment and control of helminth infections in humans. Expert Rev Anti Infect Ther 17:51–56. doi:10.1080/14787210.2018.1555241 30501436 PMC6376865

[12] Gavidia CM, Gonzalez AE, Barron EA, Ninaquispe B, Llamosas M, Verastegui MR, Robinson C, Gilman RH. 2010. Evaluation of oxfendazole, praziquantel and albendazole against cystic echinococcosis: a randomized clinical trial in naturally infected sheep. PLoS Negl Trop Dis 4:e616. doi:10.1371/journal.pntd.0000616 20186332 PMC2826409

[13] López‐Muñoz FJ. 1994. Surface of synergistic interaction between dipyrone and morphine in the PIFIR model. Drug Dev Res 33:26–32. doi:10.1002/ddr.430330105

[14] Palomares-Alonso F, Rojas-Tomé IS, Palencia Hernández G, Jiménez-Arellanes MA, Macías-Rubalcava ML, González-Maciel A, Ramos-Morales A, Santiago-Reyes R, Castro N, González-Hernández I, Rufino-González Y, Jung-Cook H. 2017. In vitro and in vivo cysticidal activity of extracts and isolated flavanone from the bark of Prunus serotina: a bio-guided study. Acta Trop 170:1–7. doi:10.1016/j.actatropica.2017.02.023 28216368

[15] Louis KS, Siegel AC. 2011. Cell viability analysis using trypan blue: manual and automated methods. Methods Mol Biol 740:7–12. doi:10.1007/978-1-61779-108-6_2 21468962

[16] Saemi Soudkolaei A, Kalidari GA, Borji H. 2021. Anthelmintic efficacy of fenbendazole and levamisole in native fowl in northern Iran. Parasites Vectors 14:104. doi:10.1186/s13071-021-04605-9 33557928 PMC7871369

[17] Holsback L, Luppi PAR, Silva CS, Negrão GK, Conde G, Gabriel HV, Balestrieri JV, Tomazella L. 2016. Anthelmintic efficiency of doramectin, fenbendazole, and nitroxynil, in combination or individually, in sheep worm control. Rev Bras Parasitol Vet 25:353–358. doi:10.1590/S1984-29612016025 27096532

[18] Cumberland WN, Fong Y, Yu X, Defawe O, Frahm N, De Rosa S. 2015. Nonlinear calibration model choice between the four and five-parameter logistic models. J Biopharm Stat 25:972–983. doi:10.1080/10543406.2014.920345 24918306 PMC4263697

[19] Palomares-Alonso F, Jung-Cook H, Pérez-Villanueva J, Piliado JC, Rodríguez-Morales S, Palencia-Hernández G, López-Balbiaux N, Hernández-Campos A, Castillo R, Hernández-Luis F. 2009. Synthesis and in vitro cysticidal activity of new benzimidazole derivatives. Eur J Med Chem 44:1794–1800. doi:10.1016/j.ejmech.2008.05.005 18582991

[20] Palomares-Alonso F, González CR, Bernad-Bernad MJ, Montiel MDC, Hernández GP, González-Hernández I, Castro-Torres N, Estrada EP, Jung-Cook H. 2010. Two novel ternary albendazole-cyclodextrin-polymer systems: dissolution, bioavailability and efficacy against Taenia crassiceps cysts. Acta Trop 113:56–60. doi:10.1016/j.actatropica.2009.09.006 19769931

[21] Mkupasi EM, Ngowi HA, Sikasunge CS, Leifsson PS, Johansen MV. 2013. Efficacy of ivermectin and oxfendazole against Taenia solium cysticercosis and other parasitoses in naturally infected pigs. Acta Trop 128:48–53. doi:10.1016/j.actatropica.2013.06.010 23806569

[22] Thomas CM, Timson DJ. 2020. The mechanism of action of praziquantel: can new drugs exploit similar mechanisms? Curr Med Chem 27:676–696. doi:10.2174/0929867325666180926145537 30259811

[23] Lacey E. 1990. Mode of action of benzimidazoles. Parasitol Today 6:112–115. doi:10.1016/0169-4758(90)90227-u 15463312

[24] Francisca P-A, Javier L-M, Guadalupe PH, Fernanda G-M, Nelly C, Helgi J-C, Iliana G-H, Susana R-TI. 2020. Cysticidal activity of praziquantel-mebendazole combination: in vitro and in vivo studies. Acta Trop 202:105238. doi:10.1016/j.actatropica.2019.105238 31669532

[25] Cederberg S, Sikasunge CS, Andersson A, Johansen MV. 2012. Short communication: in vitro efficacy testing of praziquantel, ivermectin, and oxfendazole against Taenia Solium cysts. J Parasitol Res 2012:363276. doi:10.1155/2012/363276 21785697 PMC3135050

[26] Cridge H, Lupiano H, Nipper JD, Mackin AJ, Steiner JM. 2021. Efficacy of a low-dose praziquantel and fenbendazole protocol in the treatment of asymptomatic schistosomiasis in dogs. J Vet Intern Med 35:1368–1375. doi:10.1111/jvim.16142 33955589 PMC8163111

[27] Palomares F, Palencia G, Pérez R, González-Esquivel D, Castro N, Cook HJ. 2004. In vitro effects of albendazole sulfoxide and praziquantel against Taenia solium and Taenia crassiceps cysts. Antimicrob Agents Chemother 48:2302–2304. doi:10.1128/AAC.48.6.2302-2304.2004 15155240 PMC415591

[28] Arruda GC, Da Silva ADT, Quagliato E, Maretti MA, Rossi CL. 2005. Evaluation of Taenia solium and Taenia crassiceps cysticercal antigens for the serodiagnosis of neurocysticercosis. Tropical Med Int Health 10:1005–1012. doi:10.1111/j.1365-3156.2005.01480.x 16185235

[29] Zurabian R, Aguilar-Vega L, Terrones Vargas E, Cervera Hernández ME, Willms K, Ruíz-Velasco Acosta S. 2013. In vivo albendazole treatment of Taenia crassiceps cysticerci strain WFU: proliferation, damage, and recovery. Parasitol Res 112:3961–3968. doi:10.1007/s00436-013-3589-7 24005476

[30] Gokbulut C, Bilgili A, Hanedan B, McKellar QA. 2007. Comparative plasma disposition of fenbendazole, oxfendazole and albendazole in dogs. Vet Parasitol 148:279–287. doi:10.1016/j.vetpar.2007.06.028 17673370

[31] Ngomuo AJ, Marriner SE, Bogan JA. 1984. The pharmacokinetics of fenbendazole and oxfendazole in cattle. Vet Res Commun 8:187–193. doi:10.1007/BF02214711 6495635

[32] An G, Murry DJ, Gajurel K, Bach T, Deye G, Stebounova LV, Codd EE, Horton J, Gonzalez AE, Garcia HH, Ince D, Hodgson-Zingman D, Nomicos EYH, Conrad T, Kennedy J, Jones W, Gilman RH, Winokur P. 2019. Pharmacokinetics, safety, and tolerability of oxfendazole in healthy volunteers: a randomized, placebo-controlled first-in-human single-dose escalation study. Antimicrob Agents Chemother 63:e02255-18. doi:10.1128/AAC.02255-18 30745383 PMC6437481

[33] Bach T, Galbiati S, Kennedy JK, Deye G, Nomicos EYH, Codd EE, Garcia HH, Horton J, Gilman RH, Gonzalez AE, Winokur P, An G. 2020. Pharmacokinetics, safety, and tolerability of oxfendazole in healthy adults in an open-label phase 1 multiple ascending dose and food effect study. Antimicrob Agents Chemother 64:e01018-20. doi:10.1128/AAC.01018-20 32816721 PMC7577123

